# Atrial performance in healthy subjects following high altitude exposure at 4100 m: 2D speckle-tracking strain analysis

**DOI:** 10.1007/s10554-021-02173-8

**Published:** 2021-02-05

**Authors:** Chunyan He, Chuan Liu, Shiyong Yu, Jie Yang, Xiaohan Ding, Shizhu Bian, Jihang Zhang, Jie Yu, Hu Tan, Jun Jin, Mingdong Hu, Guoming Wu, Chen Zhang, Rongsheng Rao, Lan Huang

**Affiliations:** 1 Institute of Cardiovascular Diseases of PLA, the Second Affiliated Hospital, Third Military Medical University (Army Medical University), Chongqing, 400037 China; 2 Department of Cardiology, the Second Affiliated Hospital, Third Military Medical University (Army Medical University), Chongqing, 400037 China; 3 Department of Health Care and Geriatrics, the 940th Hospital of Joint Logistics Support Force of PLA, Lanzhou, China; 4 Department of Respiratory Medicine, the Second Affiliated Hospital, Third Military Medical University (Army Medical University), Chongqing, China; 5 Department of Medical Ultrasonics, the Second Affiliated Hospital, Third Military Medical University (Army Medical University), Chongqing, 400037 China

**Keywords:** High altitude, Atrial function, Echocardiography, 2D speckle-tracking

## Abstract

High altitude (HA) exposure has been considered as a cardiac stress and might impair ventricular diastolic function. Atrial contraction is involved in ventricular passive filling, however the atrial performance to HA exposure is poorly understood. This study aimed to evaluate the effect of short-term HA exposure on bi-atrial function. Physiological and 2D-echocardiographic data were collected in 82 healthy men at sea level (SL, 400 m) and 4100 m after an ascent within 7 days. Atrial function was measured using volumetric and speckle-tracking analyses during reservoir, conduit and contractile phases of cardiac cycle. Following HA exposure, significant decreases of reservoir and conduit function indexes were observed in bi-atria, whereas decreases of contractile function indexes were observed in right atrium (RA), estimated via RA active emptying fraction (SL 41.7 ± 13.9% vs. HA 35.4 ± 12.2%, p = 0.001), strain during the contractile phase [SL 13.5 (11.4, 17.8) % vs. HA 12.3 (9.3, 15.9) %, p = 0.003], and peak strain rate during the contractile phase [SL − 1.76 (− 2.24, − 1.48) s^−1^ vs. HA − 1.57 (− 2.01, − 1.23) s^−1^, p = 0.002], but not in left atrium (LA). In conclusion, short-term HA exposure of healthy individuals impairs bi-atrial performance, mostly observed in RA. Especially, atrial contractile function decreases in RA rather than LA, which seems not to compensate for decreased ventricular filling after HA exposure. Our findings may provide a novel evidence for right-sided heart dysfunction to HA exposure.

**Supplementary Information:**

The online version of this article (10.1007/s10554-021-02173-8) contains supplementary material, which is available to authorized users.

## Introduction

An increasing number of lowlanders visit high altitude (HA) for work or leisure. However, HA exposure challenges cardiac function to meet the tissue metabolic demand for oxygen under hypoxic conditions [1]. It has been well established that the cardiac response to HA exposure presents preserved ventricular systolic function, impaired ventricular diastolic function, and elevated pulmonary arterial pressure [2]. Atrial contraction is the final component during ventricular diastole and contributes approximately 15% to 20% of stroke volume as a compensatory mechanism [3–5]. Evidence is lacking regarding atrial response to HA exposure. Until recently, Sareban et al. [6, 7] reported unchanged left atrial (LA) and enhanced right atrial (RA) contractile function after a few hours following an ascent to 4559 m. It actually takes several days to acclimate to HA conditions with cardiac output returning to normal through a higher heart rate and lower stroke volume [8]. Nevertheless, no studies to date have systematically described atrial performance under short-term HA exposure. HA exposure induces hypoxic pulmonary hypertension, and consequently increases right ventricular (RV) afterload, directly conducting to RA. Due to differences of pressure and resistance from vascular attachments, RA performance may be different from LA after HA exposure. Thus, it is of great value to evaluate the effect of short-term HA exposure on bi-atrial function, which can provide a novel insight into cardiac adaption to altitude exposure.

The atrium plays an important role in modulating ventricular filling by means of three phases: the reservoir phase during ventricular systole, the conduit phase during ventricular early diastole and the contractile phase during ventricular late diastole [9]. Doppler echocardiography has been previously used to assess relative atrial function, however it is subject to error because of angle dependence and non-specificity [10–12]. Recently, speckle-tracking echocardiography (STE) gradually supersedes Doppler imaging, which can quantify regional and global atrial myocardial deformation representing intrinsic myocardial properties [13, 14]. In this study, we aimed to investigate the effect of short-term HA exposure on bi-atrial function using STE and identify the related factors.

## Methods

### Study population and procedure

Healthy men from Han ethnicity aged 18–45 years old and permanently living below 500 m above sea level (asl) were recruited in June 2013. We excluded the subjects with the following: known cardiovascular and pulmonary disease (such as congenital heart disease, valvular disease, arrhythmia, chronic obstructive pulmonary disease, asthma), previous history of exposure to altitude above 2500 m asl in the past 6 months, and missing data or poor quality images. Finally, 82 subjects were enrolled in the analysis. The experimental protocol was registered under the Chinese Clinical Trial Registration (No: ChiCTR-RCS-12002232, http://www.chictr.org.cn). The study received approval by the Clinical Research Ethics Committee of the Third Military Medical University (Army Medical University) (NO: 2012015), in accordance with Declaration of Helsinki, and all subjects granted informed content for participation.

All subjects ascended to Litang (Sichuan, China, 4100 m asl) from Yanggongqiao (Chongqing, China, 400 m asl) by bus within 7 days. The subjects enrolled in our study underwent clinical examination and standard transthoracic echocardiography at sea level (SL, 400 m asl) and in 5 ± 2 h after arrival at 4100 m.

### Clinical examination

Clinical data recorded for all subjects included age, height, and weight. Body mass index (BMI) and body surface area (BSA) were calculated according to the customary formula [15]. Blood pressure was measured by Omron HEM-6200 (Japan) after resting for at least 5 min. Arterial pulse oxygen saturation (SpO_2_) was measured using a pulse oximeter (Nonin ONYX OR9500, USA).

### Echocardiographic image acquisition

The subjects underwent standard transthoracic echocardiography by an experienced cardiac sonographer. A commercially available CX50 ultrasound machine (Philips Ultrasound System, Andover, MA, USA) equipped with a 2.5 MHz frequency transducer was used to acquire images with a frame rate of 70–90 fps. The electrocardiogram connected to the ultrasound system recorded heart rate (HR) during the examination. All images were acquired in accordance with the recommendations of the American Society of Echocardiography [16]. The echocardiographic images were saved digitally and analyzed offline by two independent sonographer blinded to the data, using a commercially available workstation (QLAB version 10.5, Philips Healthcare, Andover, MA, USA).

### Two-dimensional and Doppler echocardiography

Ventricular area and volume were measured during ventricular end-systole and end-diastole by two-dimensional (2D) echocardiography to calculate left ventricular ejection fraction (LVEF) [17] and RV fractional area change (FAC) [18]. Atrial volumes were obtained by tracing the atrial endocardium using Simpson’s method. Atrial maximal volume (V_max_) was obtained in end-systole at the onset of mitral/tricuspid valve opening, atrial minimal volume (V_min_) was obtained at the onset of mitral/tricuspid valve closure by QRS complex of ECG, and atrial pre-systolic volume (V_pre-A_) was obtained preceding the P wave. All volume measurements were indexed to the BSA, and used to calculate atrial phasic emptying fractions, including total (EFtot), passive (EFpass) and active (EFact) components [10, 19].

From the pulsed-wave Doppler echocardiography of blood flow velocities at mitral and tricuspid valves, the peak early diastolic E-wave velocity, peak late diastolic A-wave velocity and peak tricuspid regurgitant (TR) velocity (TRV) were acquired. Systolic pulmonary arterial pressure (sPAP) was calculated as follows: 4 × TRV ^2^ + 5 mmHg (an estimated central venous pressure) [20, 21]. From pulsed-wave tissue Doppler images of mitral and tricuspid annuli, ventricular systolic S′ velocity, early diastolic E′ velocity, and late diastolic A′ velocity at the septal and lateral walls were measured.

### Speckle-tracking echocardiography

**Fig. 1:**
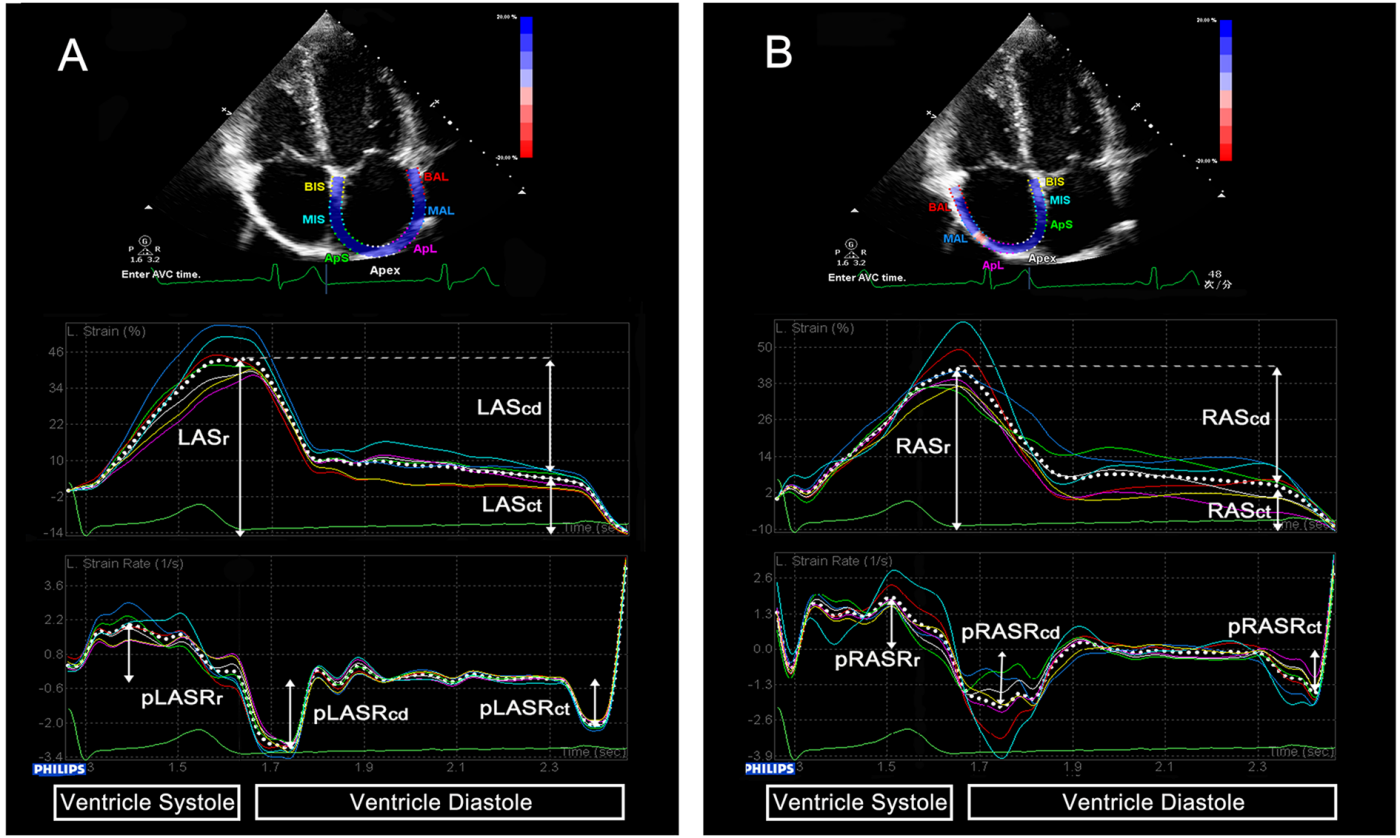
Two-dimensional speckle-tracking echocardiographic assessment of atrial function. Measurement of left atrial (**A**) and right atrial (**B**) reservoir, conduit and contractile function by strain and strain rate curves. *LA* left atrial, *RA* right atrial, *Sr* strain during the reservoir phase, *Scd* strain during the conduit phase, *Sct* strain during the contractile phase, *pSRr* peak strain rate during the reservoir phase, *pSRcd* peak strain rate during the conduit phase, *pSRct* peak strain rate during the contractile phase

Atrial phasic strain and strain rate (SR) were obtained by 2D-STE. A 3-point click on the endocardial surface of the atrium and then the endocardial-epicardial borders were traced automatically by the system in four-chamber view. The optimized region of interest was manually adjusted for adequate speckle tracking. The software divided the region into seven segments, and generated strain and SR curves for each myocardial segment. The frame at QRS wave onset was used as the first reference frame. The atrial strain and peak SR during ventricular systole, early and late diastole were measured to evaluate atrial reservoir (Sr, pSRr), conduit (Scd, pSRcd), and contractile (Sct, pSRct) function respectively according to the recommendations of the European Society of Cardiology [22] as in Fig. 1. The noninvasive atrial stiffness index was calculated as the ratio of average E/E′ to Sr [23].

### Pulmonary function test

Pulmonary function test was conducted in 42 of these subjects who were randomly assigned. Spirometry was performed with a portable spirometer (Minato AS-507; Minato Medical Science Co., Ltd., Osaka, Japan) in compliance with standard techniques [24]. Pulmonary function measurements included forced vital capacity, forced expiratory volume in the first second, and maximum mid-expiratory flow. Subsequently, residual volume and total lung capacity were calculated.

### Statistical analysis

Statistical analysis was performed using SPSS 22.0 (IBM Corp., Armonk, NY, USA) and GraphPad Prism 7.0 (Inc., La Jolla, USA). The normality of continuous variables was tested by the Kolmogorov–Smirnov test. Continuous variables with a normal distribution were expressed as the mean ± standard deviation (SD), Continuous variables with a non-normal distribution were expressed as the median (interquartile range), and categorical variables were expressed as the counts and proportions. Paired-t test or Wilcoxon matched-pairs signed rank test were used for the comparison of continuous variables, as appropriate. Pearson’s correlation was performed to analyze relationships among normally distributed continuous variables, and Spearman’s correlation was used for non-normally distributed statistics. A p value < 0.05 was defined as statistically significant.

Intra- and inter-observer variabilities were assessed in 20 randomly selected subjects. Inter-observer variability was performed by two independent observers, and intra-observer variability was performed by the same observer at least 1 month apart. Both the intra-observer and inter-observer variabilities were tested using the intra-class correlation coefficient (ICC) by Cronbach’s α.

## Results

### The effect of HA exposure on cardiac function

The mean age of the subjects was 20 (19–21) years old. The cardiac response to short-term HA exposure was presented in Table 1. Following HA exposure, increases in systolic blood pressure, diastolic blood pressure and HR, and decrease in SpO_2_ were observed. For LV parameters, unchanged EDV index and significantly decreased ESV index, thus, increased LVEF were observed. For RV parameters, significantly decreased EDA index while unchanged ESA index, and consequently decreased RV FAC were recorded at HA. Additionally, both mitral and tricuspid E/A decreased after HA exposure. As expected, the proportion of subjects with tricuspid regurgitation was greater at HA. Thus, sPAP significantly increased after HA exposure. From pulsed-wave Doppler echocardiography (Table 2), no significant change was observed in mitral and tricuspid S' and E'. However, decreased A' was observed at tricuspid annulus, but not at mitral annulus. Besides, E/E' decreased at both mitral and tricuspid valves.

**Table 1:** Physiological parameters and conventional echocardiographic parameters at sea level and high altitude

Variables	Sea level (n = 82)	High altitude (n = 82)	P-value
Physiological parameters			
Age (years)	20.0 (19.0, 21.0)	–	
Height (m)	1.72 ± 0.04	–	
Weight (kg)	61.9 ± 6.2	–	
BMI (kg/m^2^)	20.9 ± 1.7	–	
BSA (m^2^)	1.69 ± 0.10	–	
SpO_2_ (%)	98.0 (97.0, 98.0)	89.0 (88.0, 91.0)	** < 0.001**
Heart rate (bpm)	66.0 (59.8, 75.0)	72.5 (64.0, 81.0)	** < 0.001**
SBP (mmHg)	112.6 ± 10.1	120.0 ± 11.1	** < 0.001**
DBP (mmHg)	68.8 ± 8.5	78.3 ± 9.5	** < 0.001**
Left heart parameters			
LVEDV index (ml/m^2^)	57.5 (48.6, 66.7)	53.1 (44.5, 62.1)	0.051
LVESV index (ml/m^2^)	22.5 ± 8.2	19.0 ± 6.4	** < 0.001**
LVEF (%)	61.0 ± 11.0	65.0 ± 9.0	**0.007**
Mitral E-wave (cm/s)	98.1 (90.0, 108.5)	78.9 (70.0, 88.0)	** < 0.001**
Mitral A-wave (cm/s)	54.7 ± 12.9	50.2 ± 11.3	**0.016**
Mitral E/A	1.79 (1.47, 2.20)	1.59 (1.34, 1.87)	** < 0.001**
LA diameter (cm)	3.63 ± 0.38	3.33 ± 0.45	** < 0.001**
LAEDA (cm^2^)	11.7 (10.3, 12.9)	11.1 (9.6, 12.8)	**0.039**
LAESA (cm^2^)	5.98 ± 1.51	6.03 ± 1.56	0.820
Right heart parameters			
RVEDA index (cm^2^/m^2^)	13.2 ± 2.3	12.5 ± 2.2	**0.009**
RVESA index (cm^2^/m^2^)	7.26 ± 1.31	7.37 ± 1.45	0.471
RV FAC (%)	44.8 ± 4.1	41.1 ± 4.0	** < 0.001**
Tricuspid E-wave (cm/s)	73.6 (63.6, 81.7)	61.8 (53.5, 68.1)	** < 0.001**
Tricuspid A-wave (cm/s)	37.5 ± 9.1	34.8 ± 8.7	**0.038**
Tricuspid E/A	1.98 (1.58, 2.47)	1.78 (1.41, 2.33)	**0.020**
RA diameter (cm)	3.95 ± 0.53	3.86 ± 0.55	0.208
RAEDA (cm^2^)	11.8 ± 2.3	11.1 ± 2.4	**0.011**
RAESA (cm^2^)	6.00 (5.10, 7.40)	6.65 (5.70, 7.50)	**0.013**
TR (n (%)	51 (62.2%)	65 (79.3%)	**0.004**
TR velocity (cm/s)	215.9 ± 31.1	247.9 ± 40.2	** < 0.001**
sPAP (mmHg)	24.0 ± 5.4	30.2 ± 8.0	** < 0.001**

Data are expressed as mean ± SD, median (interquartile range), or n (%). Bold values indicate statistically significant. *BMI* body mass index, *BSA* body surface area, *SpO2*, arterial pulse oxygen saturatison, *SBP* Systolic blood pressure, *DBP* Diastolic blood pressure, *LV* left ventricle, *EDV* end-diastolic volume, *ESV* end-systolic volume, *LVEF* Left ventricular ejection fraction, *E-wave* peak early diastolic annular inflow velocity, *A-wave* peak late diastolic annular inflow velocity, *LA* left atrium, *EDA* end-diastolic area, *ESA* end-systolic area, *RV* right ventricle, *FAC* fractional area change, *RA* right atrium, *TR* tricuspid regurgitation, *sPAP* systolic pulmonary arterial pressure

**Table 2:** Tissue velocities at the mitral and tricuspid annuli at sea level and high altitude

	Mitral annulus (n = 82)			Tricuspid annulus (n = 82)		
	Sea level	High altitude	P-value	Sea level	High altitude	P-value
Tissue velocities measured at the septal annulus						
S′ (cm/s)	10.1 (8.8, 10.9)	9.77 (8.23, 11.08)	0.662	9.37 (8.48, 10.26)	8.58 (8.15, 9.54)	**0.032**
E′ (cm/s)	14.2 (13.1, 15.9)	12.6 (11.1, 14.0)	** < 0.001**	14.5 (13.0, 16.3)	12.5 (11.4, 13.9)	** < 0.001**
A′ (cm/s)	9.00 (7.55, 10.67)	8.18 (7.21, 9.12)	0.168	7.89 (6.61, 9.16)	7.76 (6.35, 8.55)	**0.011**
E/E′	6.89 (6.31, 7.52)	6.48 (5.09, 7.70)	0.317	5.23 (4.32, 6.08)	4.93 (4.21, 5.93)	0.878
Tissue velocities measured at the lateral annulus						
S′ (cm/s)	12.8 (10.9, 15.5)	13.2 (11.1, 15.3)	0.457	14.9 (13.4, 16.6)	13.9 (12.3, 16.0)	0.068
E′ (cm/s)	19.4 (17.0, 21.9)	20.4 (16.8, 23.0)	0.228	15.9 (14.1, 18.4)	15.8 (13.7, 18.1)	0.540
A′ (cm/s)	8.66 (6.83, 10.42)	8.10 (6.63, 9.17)	0.095	10.4 (9.0, 13.2)	9.54 (7.31, 11.99)	** < 0.001**
E/E′	5.20 (4.10, 5.89)	3.79 (3.11, 5.05)	** < 0.001**	4.79 (3.68, 5.64)	3.85 (3.20, 4.89)	**0.003**
Average tissue velocities measured at the septal and lateral annuli						
S′ (cm/s)	11.5 (10.5, 12.9)	11.7 (9.7, 13.0)	0.747	12.4 (11.2, 13.9)	11.9 (10.4, 14.1)	0.708
E′ (cm/s)	17.1 (15.6, 18.9)	16.4 (14.6, 19.2)	0.341	15.3 (14.0, 16.8)	14.7 (12.9, 16.0)	0.092
A′ (cm/s)	8.93 (7.63, 10.23)	8.38 (7.13, 9.24)	0.064	9.42 (8.09, 11.40)	8.65 (7.51, 10.26)	**0.001**
E/E′	5.95 (4.91, 6.59)	4.70 (3.85, 5.85)	**0.001**	5.02 (3.95, 5.75)	4.07 (3.41, 5.01)	**0.008**

Data are expressed as mean ± SD or median (interquartile range). Bold values indicate statistically significant. *S′* peak ventricular systolic tissue velocity, *E′* peak ventricular early diastolic tissue velocity, *A′* peak ventricular late diastolic tissue velocity, *E/E′* the ratio between peak ventricular early diastolic annular inflow velocity and peak ventricular early diastolic tissue velocity

### The effect of HA exposure on bi-atrial phasic volumetric and strain parameters

**Fig. 2:**
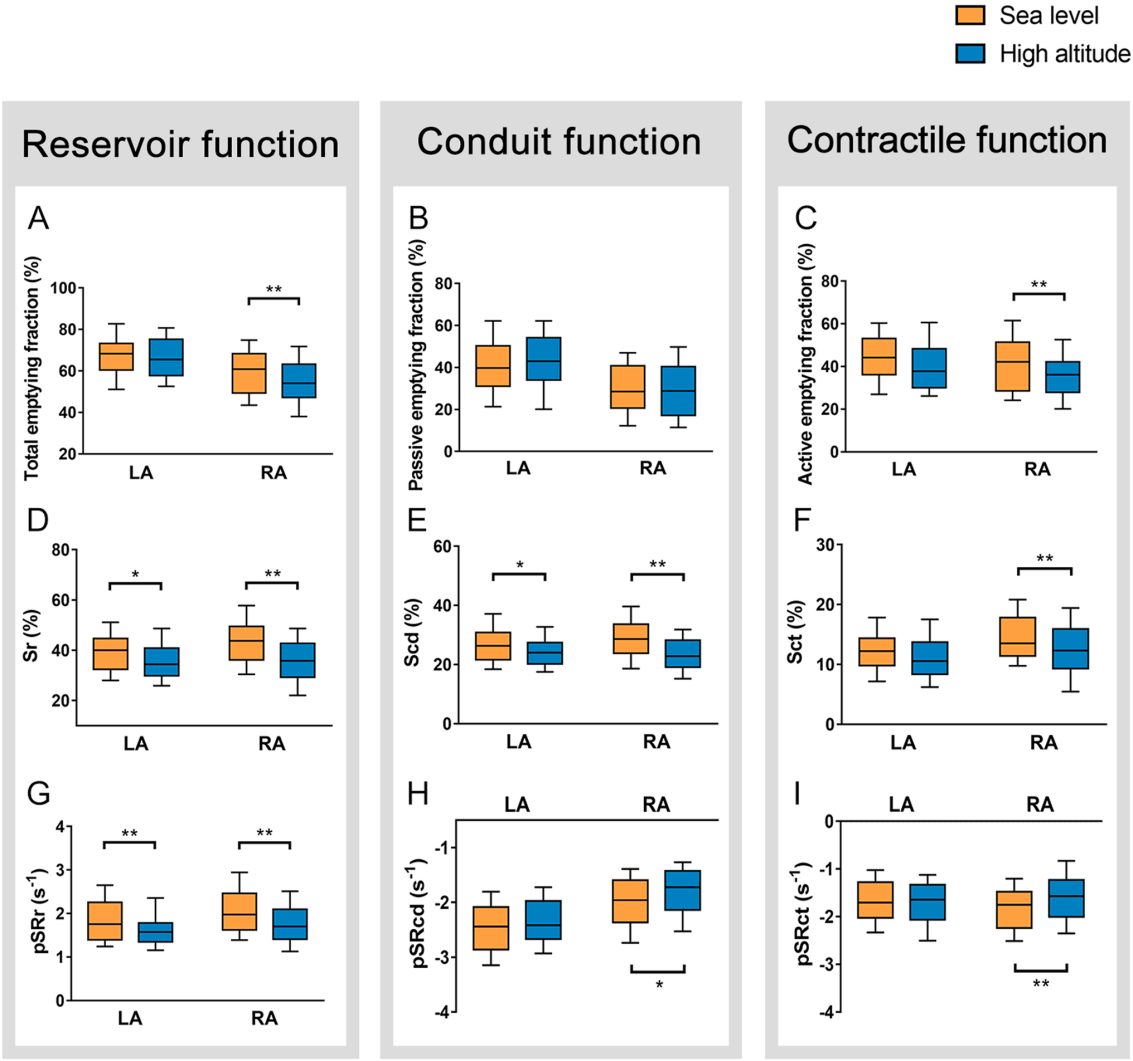
Comparisons of atrial phasic function between left and right atria under high altitude exposure. *p < 0.05; **p < 0.01. Abbreviations as in Fig. 1

The comparisons of atrial phasic function assessed by volume and speckle-tracking analysis between before and after HA exposure were presented in Table 3. Significant decreases were observed in the LAV_max_ and LAV_pre-A_, but not in RA volume indexes. However, a trend towards increase was observed in RAVmin after HA exposure. Besides, significant decreases were demonstrated in RAEFtot (SL 59.7 ± 11.6% vs. HA 54.5 ± 12.3%, p = 0.001), RA expansion index [SL 154.8 (97.5, 215.9) % vs. HA 117.3 (89.4, 171.7) %, p = 0.006], and RAEFact (SL 41.7 ± 13.9% vs. HA 35.4 ± 12.2%, p = 0.001) after ascending to HA, but not in LA indexes. Table 4 showed highly significant decreases in RA strain during reservoir (SL 43.5 ± 10.0% vs. HA 35.8 ± 9.5%, p < 0.001), conduit (SL 29.1 ± 7.5% vs. HA 23.3 ± 6.8%, p < 0.001) and contractile phases [SL 13.5 (11.4, 17.8)% vs. HA 12.3 (9.3, 15.9) %, p = 0.003], and LA strain during reservoir and conduit phases after HA exposure. Besides, for SR, RA decreased during reservoir [SL 1.98 (1.62, 2.47) s^−1^ vs. HA 1.70 (1.41, 2.09) s^−1^, p < 0.001], conduit [SL − 1.96 (− 2.37, − 1.59) s^−1^ vs. HA − 1.72 (− 2.14, − 1.42) s^−1^, p = 0.037], and contractile phases [SL − 1.76 (− 2.24, − 1.48) s^−1^ vs. HA − 1.57 (− 2.01, − 1.23) s^−1^, p = 0.002]. However, LA SR only decreased during reservoir phase (Fig. 2). Additionally, the bi-atrial stiffness indexes were not affected by HA exposure.

**Table 3:** Bi-atrial phasic volume indexes and emptying fractions at sea level and high altitude

	Left atrium (n = 82)			Right atrium (n = 82)		
	Sea level	High altitude	P-value	Sea level	High altitude	P-value
Maximal volume index (ml/m^2^)	18.4 (15.7, 22.6)	15.4 (12.7, 20.3)	**0.003**	18.5 ± 5.5	17.8 ± 5.5	0.423
Minimal volume index (ml/m^2^)	5.92 (3.90, 8.40)	5.07 (3.45, 7.74)	0.236	7.03 (5.35, 9.30)	7.81 (6.05, 10.07)	0.059
Pre-systolic volume index (ml/m^2^)	11.27 (8.31, 13.85)	9.28 (6.36, 12.11)	**0.007**	12.5 (9.54, 15.4)	12.5 (10.0, 16.3)	0.767
Reservoir function						
Total emptying volume index (ml/m^2^)	12.3 (10.3, 15.2)	10.4 (8.6, 13.1)	** < 0.001**	10.86 (8.44, 13.87)	9.23 (6.99, 11.44)	**0.006**
Total emptying fraction (%)	67.0 ± 10.7	66.2 ± 11.3	0.626	59.7 ± 11.6	54.5 ± 12.3	**0.001**
Expansion index (%)	215.6 (152.8, 274.0)	189.4 (136.8, 302.5)	0.699	154.8 (97.5, 215.9)	117.3 (89.4, 171.7)	**0.006**
Conduit function						
Passive emptying volume index (ml/m^2^)	7.61 ± 2.99	7.06 ± 2.91	0.181	5.06 (3.02, 7.73)	4.85 (3.19, 7.02)	0.267
Passive emptying fraction (%)	40.5 ± 14.2	42.8 ± 14.2	0.285	30.2 ± 13.4	29.3 ± 14.7	0.664
Contractile function						
Active emptying volume index (ml/m^2^)	4.31 (3.37, 6.44)	3.16 (2.42, 4.73)	**0.001**	4.45 (3.39, 6.97)	4.08 (2.76, 5.59)	**0.029**
Active emptying fraction (%)	44.2 (36.2, 53.1)	37.7 (30.0, 48.3)	0.073	41.7 ± 13.9	35.4 ± 12.2	**0.001**

Data are expressed as mean ± SD, or median (interquartile range). Bold values indicate statistically significant

**Table 4:** Bi-atrial strain and strain rate by speckle tracking echocardiography at sea level and high altitude

	Left atrium (n = 82)			Right atrium (n = 82)		
	Sea level	High altitude	P-value	Sea level	High altitude	P-value
Reservoir function						
Sr (%)	40.0 (33.4, 44.7)	34.3 (29.9, 40.8)	**0.016**	43.5 ± 10.0	35.8 ± 9.5	** < 0.001**
pSRr (s^−1^)	1.76 (1.40, 2.26)	1.58 (1.35, 1.78)	**0.004**	1.98 (1.62, 2.47)	1.70 (1.41, 2.09)	** < 0.001**
Conduit function						
Scd (%)	26.3 (21.6, 30.8)	24.0 (20.2, 27.4)	**0.015**	29.1 ± 7.5	23.3 ± 6.8	** < 0.001**
pSRcd (s^−1^)	− 2.44 (− 2.87, − 2.09)	− 2.42 (− 2.67, − 1.98)	0.105	− 1.96 (− 2.37, − 1.59)	− 1.72 (− 2.14, − 1.42)	**0.037**
Contractile function						
Sct (%)	12.2 (9.8, 14.4)	10.6 (8.3, 13.7)	0.067	13.5 (11.4, 17.8)	12.3 (9.3, 15.9)	**0.003**
pSRct (s^−1^)	− 1.71 (− 2.03, − 1.28)	− 1.65 (− 2.07, − 1.33)	0.972	− 1.76 (− 2.24, − 1.48)	− 1.57 (− 2.01, − 1.23)	**0.002**
Atrial stiffness index	0.28 (0.24, 0.35)	0.29 (0.24, 0.36)	0.632	0.23 (0.17, 0.29)	0.24 (0.19, 0.30)	0.462

Data are expressed as mean ± SD, or median (interquartile range). Bold values indicate statistically significant. *Sr* strain during the reservoir phase, *Scd* strain during the conduit phase, *Sct* strain during the contractile phase, *pSRr* peak strain rate during the reservoir phase, *pSRcd* peak strain rate during the conduit phase, *pSRct* peak strain rate during the contractile phase

### Effect of SpO_2_ and TR on RA contractile function at HA

**Fig. 3:**
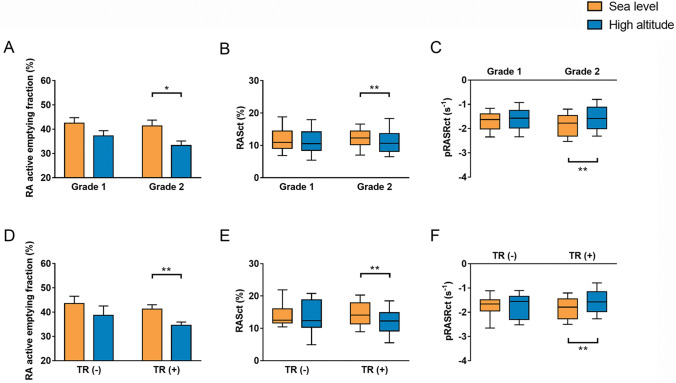
Effect of oxygen saturation and tricuspid regurgitation on right atrial contractile function at high altitude. The subjects were stratified into two groups according to the decline of SpO_2_ (**A**, **B** and **C**) or the presence of TR after high altitude exposure (**D**, **E** and **F**), respectively. *p < 0.05; **p < 0.01. SpO_2_, arterial pulse oxygen saturation; *TR* tricuspid regurgitation. Other abbreviations as in Fig. 1

The subjects were stratified according to the decline of SpO_2_ after HA exposure. As presented in Fig. 3, significant decreases of RAEFact, RASct and pRASRct were observed in the group with higher decline of SpO_2_ (p < 0.05), but not in the lower group. Additionally, according to previous study that any increase in TR was associated with progressive increase in pressure of right-sided heart [25], the subjects were stratified according to the presence of TR at HA. As presented in Fig. 3, significant decreases of RAEFact, RASct and pRASRct were observed in subjects with TR (p < 0.05) after HA exposure, but not in subjects without TR.

### Correlations of RA phasic function with other parameters

The correlations of RA phasic function with physiological and other echocardiographic parameters after HA exposure were illustrated in Supplementary Table 1. ΔRAEFtot (r = 0.22, p = 0.047) showed a positive correlation with age. ΔRASr (r = − 0.30, p = 0.007), ΔRAScd (r = − 0.28, p = 0.011) and ΔpRASRr (r = − 0.31, p = 0.005) showed negative correlations with BMI. ΔRASr (r = − 0.26, p = 0.018) and ΔpRASRcd (r = 0.26, p = 0.020) showed significant correlations with ΔSpO_2_. ΔRAEFact (r = − 0.24, p = 0.032) showed negative correlations with Δtricuspid E/A. ΔRAEFtot (r = 0.32, p = 0.042) and ΔRAEFact (r = 0.39, p = 0.010) showed significantly positive correlations with ΔsPAP. Additionally, the correlations of RA contractile function with pulmonary function after HA exposure were presented in Supplementary Table 2. However, no significant correlations of RAEFact, RASct and pRASRct with any pulmonary function indexes were observed after HA exposure (p > 0.05).

### Reproducibility

Intra- and inter-observer ICC was 0.84 (p < 0001) and 0.78 (p = 0001) for LASr, and 0.89 (p < 0001) and 0.80 (p < 0001) for RASr, respectively. Intra- and inter-observer ICC for other atrial strain and SR were presented in Supplementary Table 3. All measurements showed excellent or good reproducibility.

## Discussion

**Fig. 4:**
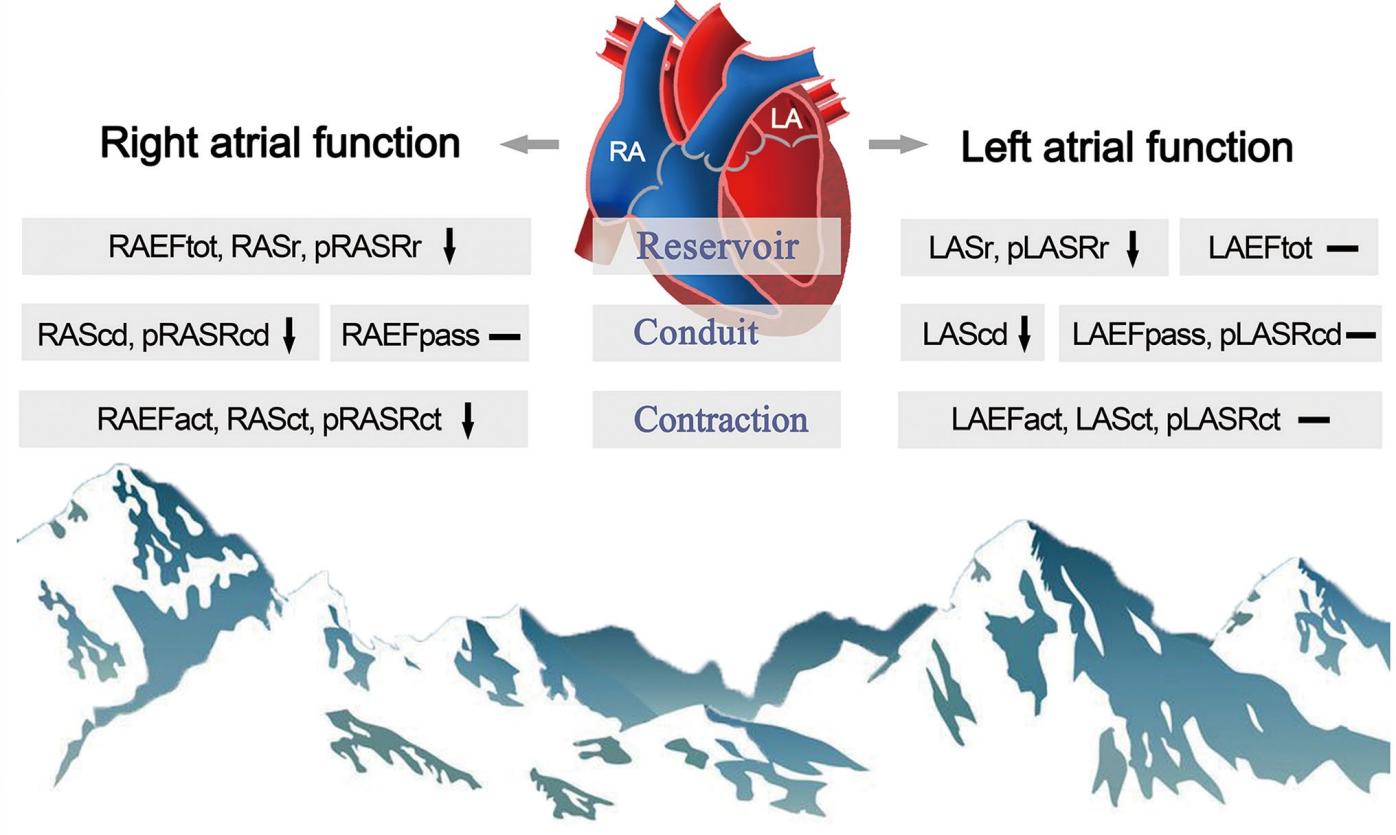
Summarizing illustration. Short-term HA exposure impairs bi-atrial performance, mostly observed in RA. Especially, atrial contractile function decreases in RA rather than LA. *EFtot* total emptying fraction, *EFpass* passive emptying fraction, *EFact* active emptying fraction. Other abbreviations as in Fig. 1

To our knowledge, this is the first study to comprehensively evaluate the effect of short-term HA exposure on bi-atrial performance using STE. The major findings of our study were that HA exposure led to decreases in bi-atrial function, mostly in RA. After short-term HA exposure, decreased reservoir and conduit functions were observed in bi-atria, whereas decreased contractile function was observed in RA (estimated via RAEFact, RASct and pRASRct) rather than LA (Fig. 4).

In the present study, we observed increased LV systolic function, slightly decreased RV systolic function and altered diastolic filling pattern in both ventricles under HA exposure as previously described [12, 26, 27]. Among those physiological responses, it has been well recognized that LV systolic function adapts well to HA conditions, nevertheless RV failed as a consequence of HA-induced higher pulmonary arterial pressure [27]. Additionally, our results suggested that LV and RV intrinsic relaxation seemed unaffected by hypoxia since ventricular early filling (estimated via E′) remained unchanged after short-term HA exposure.

Regarding ventricular passive filling, atrial contraction has been commonly regarded as enhanced to overcome HA exposure-induced ventricular diastolic dysfunction in previous studies using Doppler echocardiography, which actually can’t reflect the real atrial properties [11, 28]. Recently, Sareban et al. [6, 7] presented a different perspective that LA contraction did not change, however, RA contraction increased in a few hours after a rapid ascending to HA assessed by STE. However, in the present study, we obtained a novel finding that RA contraction decreased after short-term HA exposure, which seemed not to compensate for decreased ventricular filling. Previous studies have validated that volume and strain derived parameters were preload-dependent in different degrees, of which SR appeared to be less preload-dependent [29, 30]. Indeed, Robach et al. [31] documented that plasma volume decreased within 1–3 days and fell by 13.6% after 7 days at 4350 m. Similarly, the decreased mitral and tricuspid E/E' from our data implied the loss of plasma volume after short-term HA exposure, due to its sensitivity to the changes of preload [32]. Therefore, in the present study, SR could better reflect the real atrial response to HA exposure. Besides measurement methods, the discrepancies between the present study and previous findings might be explained by differences in race, exposure duration and physical activity.

As well recognized, cardiac adaptation to HA is a comprehensive consequence of hypoxia, pulmonary vasoconstriction, sympathetic activation and hypovolemia [1, 33, 34]. Additionally, it should be acknowledged that atrium interacts with ventricle throughout the cardiac cycle. Accordingly, atrial performance under HA exposure might be multiply affected by decreased energy supply and preload, increased afterload, and altered ventricular mechanics. In this study, the subgroup analyses have clarified our hypothesis that decreased RA contractile function was linked with hypoxia and pulmonary hypertension, but not pulmonary function. As generally known, hypoxia is the initial determinant of cardiopulmonary response to high altitude and previous studies have validated hypoxia alone reduced atrial contractility [35, 36]. Additionally, the presence of TR after HA exposure was majorly secondary to hypoxic pulmonary hypertension, which caused RV excessive afterload, and ultimately conducted to RA, seeming to be the common mechanism underlying decreased RV contractility at HA. RA is a thin-wall chamber and works at lower pressure than LA under physiological condition. We speculate that it is hard for RA to adapt HA-induced pressure overload, which should be responsible for the vulnerability of RA under HA conditions. However, although HA exposure induced increased ventilation, it seemed to have little effect on atrial function. Moreover, the correlation analyses might provide additional implications as age-and BMI-related changes in RA function after ascending to HA, which need enroll larger population to verify.

Ventricular adaptation at HA has been well described, but the studies on atrial response to HA is scarce. Our findings demonstrated that short-term HA exposure impaired RA contractile function, which could provide novel evidence for HA-induced RV dysfunction. The impairment of RA function in the early stage after HA exposure might develop into HA heart disease, however whether it persists or is reversible needs longer follow-up to be revealed. Additionally, it has been widely assessed that RA function was sensitive and valuable to predict exercise capacity and clinical outcomes in non-HA-induced pulmonary arterial hypertension [37–39]. Indeed, physiological adaptation to high altitude has long been recognized as hypoxic pulmonary hypertension and reduced exercise capacity, especially in trekker and mountaineer. Accordingly, the impaired RA function based on this study might be linked with limited exercise capacity at HA, and individuals with worse RA function might need to reduce physical activity and even exposure duration to avoid HA related diseases. Our findings might indicate a new approach to assess and improve HA acclimatization but remains to be determined.

### Limitations

Several limitations of this study should be acknowledged. The observational study was carried out in healthy adult males, thus, our results probably are not applicable to other populations. Therefore, additional populations should be included in further studies to confirm the present results. Although 2D-STE derived quantitative assessment of atrial function is feasible and sensitive, invasive cardiac catheterization and 3D echocardiography are warranted for comparison with our results. Finally, larger-scale studies and longer follow-up are needed to explore cardiac adaption to hypoxia exposure and its clinical relevance.

## Conclusion

For the first time, we demonstrated that bi-atrial performance decreased following short-term HA exposure, mostly observed in RA. Especially, short-term HA exposure of healthy individuals decreased RA contractile function rather than LA, not compensating for decreased ventricular filling. Our findings may provide an important evidence to understand cardiac response to HA exposure.

## Supplementary Information

Below is the link to the electronic supplementary material.

Electronic supplementary material 1 (DOCX 35 kb)
