# Supplementary material for: Atrial performance in healthy subjects following high altitude exposure at 4100 m: 2D speckle-tracking strain analysis
Source: Int J Cardiovasc Imaging. 2021 Feb 5;37(6):1891–902. doi: 10.1007/s10554-021-02173-8 (PMC8255257; doi:10.1007/s10554-021-02173-8)
Supplement: Supplementary file 1 — Electronic supplementary material 1 (DOCX 35 kb) [file 10554_2021_2173_MOESM1_ESM.docx]

**Supplementary Table 1. Correlations of RA phasic function with physiological and echocardiographic parameters.**

|  | Age | |  | BMI | |  | ΔHeart rate | |  | ΔSpO2 | |  | ΔFAC | |  | Δtricuspid E/A | |  | Δtricuspid E/E' | |  | ΔsPAP | |
| --- | --- | --- | --- | --- | --- | --- | --- | --- | --- | --- | --- | --- | --- | --- | --- | --- | --- | --- | --- | --- | --- | --- | --- |
|  | r | P-value |  | r | P-value |  | r | P-value |  | r | P-value |  | r | P-value |  | r | P-value |  | r | P-value |  | r | P-value |
| ΔRAEFtot, % | 0.22 | **0.047** |  | -0.22 | **0.047** |  | 0.20 | 0.066 |  | -0.20 | 0.073 |  | -0.13 | 0.247 |  | -0.17 | 0.118 |  | -0.07 | 0.565 |  | 0.32 | **0.042** |
| ΔRAEFpass, % | 0.19 | 0.093 |  | -0.02 | 0.882 |  | 0.11 | 0.343 |  | -0.22 | **0.043** |  | 0.02 | 0.828 |  | -0.03 | 0.804 |  | 0.04 | 0.718 |  | -0.07 | 0.660 |
| ΔRAEFact, % | 0.11 | 0.349 |  | -0.21 | 0.054 |  | 0.13 | 0.240 |  | -0.01 | 0.935 |  | -0.15 | 0.195 |  | -0.24 | **0.032** |  | -0.08 | 0.522 |  | 0.39 | **0.010** |
| ΔRASr, % | 0.11 | 0.359 |  | -0.30 | **0.007** |  | 0.03 | 0.811 |  | -0.26 | **0.018** |  | 0.00 | 0.993 |  | -0.12 | 0.290 |  | -0.08 | 0.476 |  | 0.17 | 0.285 |
| ΔRAScd, % | 0.12 | 0.311 |  | -0.28 | **0.011** |  | -0.01 | 0.945 |  | -0.17 | 0.122 |  | -0.10 | 0.375 |  | -0.04 | 0.718 |  | -0.07 | 0.559 |  | 0.09 | 0.596 |
| ΔRASct, % | 0.05 | 0.676 |  | -0.14 | 0.198 |  | 0.06 | 0.580 |  | -0.19 | 0.094 |  | 0.17 | 0.137 |  | -0.19 | 0.081 |  | -0.06 | 0.592 |  | 0.24 | 0.132 |
| ΔpRASRr, s^-1^ | 0.03 | 0.782 |  | -0.31 | **0.005** |  | 0.01 | 0.944 |  | -0.02 | 0.840 |  | 0.13 | 0.259 |  | -0.16 | 0.142 |  | 0.03 | 0.833 |  | 0.30 | 0.053 |
| ΔpRASRcd, s^-1^ | -0.19 | 0.094 |  | 0.24 | **0.036** |  | -0.14 | 0.204 |  | 0.26 | **0.020** |  | 0.14 | 0.217 |  | 0.19 | 0.089 |  | 0.08 | 0.492 |  | -0.15 | 0.357 |
| ΔpRASRct, s^-1^ | -0.06 | 0.,620 |  | -0.02 | 0.870 |  | -0.19 | 0.088 |  | 0.18 | 0.098 |  | -0.01 | 0.904 |  | 0.18 | 0.106 |  | 0.03 | 0.792 |  | -0.03 | 0.877 |

Pearson’s correlation analysis was performed with normally distributed continuous statistics, or Spearman’s correlation analysis with non-normally statistics. Δ, difference of the value between at sea level and high altitude; RA, right atrium; EFtot, total emptying fraction; EFpass, passive emptying fraction; EFact, active emptying fraction; Sr, strain during the reservoir phase; Scd, strain during the conduit phase; Sct, strain during the contractile phase; pSRr, peak strain rate during the reservoir phase; pSRcd, peak strain rate during the conduit phase; pSRct, peak strain rate during the contractile phase; BMI, body mass index; SpO2, arterial pulse oxygen saturation; FAC, fractional area change; E/A, ratio of peak early to late diastolic annular inflow velocity; E/E', ratio of peak early diastolic annular inflow velocity to peak ventricular early diastolic tissue velocity; sPAP, systolic pulmonary arterial pressure.

**Supplementary Table 2. Correlations of RA contractile function with pulmonary function after high altitude exposure.**

|  |  | FVC | |  | FEV1 | |  | FEV1/FVC | |  | MMEF | |  | RV | |  | TLC | |
| --- | --- | --- | --- | --- | --- | --- | --- | --- | --- | --- | --- | --- | --- | --- | --- | --- | --- | --- |
|  |  | r | P-value |  | r | P-value |  | r | P-value |  | r | P-value |  | r | P-value |  | r | P-value |
| Pulmonary function at high altitude | | | | | | | | | | | | | | | | | | |
| RAEFact, % |  | 0.03 | 0.844 |  | 0.02 | 0.915 |  | 0.03 | 0.844 |  | -0.02 | 0.900 |  | -0.02 | 0.891 |  | -0.07 | 0.670 |
| RASct, % |  | -0.09 | 0.575 |  | -0.04 | 0.799 |  | -0.09 | 0.575 |  | 0.09 | 0.590 |  | 0.07 | 0.683 |  | -0.09 | 0.574 |
| pRASRct, s^-1^ |  | -0.08 | 0.637 |  | -0.04 | 0.791 |  | -0.08 | 0.637 |  | 0.04 | 0.826 |  | 0.03 | 0.867 |  | -0.02 | 0.894 |
| ΔPulmonary function | | | | | | | | | | | | | | | | | | |
| ΔRAEFact, % |  | -0.09 | 0.569 |  | -0.02 | 0.912 |  | 0.13 | 0.396 |  | 0.00 | 0.993 |  | 0.03 | 0.858 |  | 0.05 | 0.748 |
| ΔRASct, % |  | -0.21 | 0.192 |  | -0.22 | 0.157 |  | -0.04 | 0.784 |  | -0.06 | 0.712 |  | 0.00 | 0.999 |  | -0.05 | 0.738 |
| ΔpRASRct, s^-1^ |  | -0.18 | 0.247 |  | -0.06 | 0.713 |  | 0.17 | 0.295 |  | 0.07 | 0.682 |  | -0.05 | 0.754 |  | 0.03 | 0.852 |

FVC, forced vital capacity; FEV1, forced expiratory volume in the first second; MMEF, maximum mid-expiratory flow; RV, residual volume; TLC, total lung capacity. Other abbreviations as in Supplementary Table 1.

**Supplementary Table 3. ICC analysis of intra- and inter-observer variations for bi-atrial strain and strain rate.**

| **Variables** | **Intra-observer variation** | | |  | **Inter-observer variation** | | |
| --- | --- | --- | --- | --- | --- | --- | --- |
|  | **ICC** | **95%CI** | **P-value** |  | **ICC** | **95%CI** | **P-value** |
| LASr, % | 0.84 | 0.59-0.94 | <0.001 |  | 0.78 | 0.44-0.91 | 0.001 |
| LAScd, % | 0.94 | 0.84-0.98 | <0.001 |  | 0.90 | 0.75-0.96 | <0.001 |
| LASct, % | 0.82 | 0.55-0.93 | <0.001 |  | 0.82 | 0.55-0.93 | <0.001 |
| pLASRr, s^-1^ | 0.96 | 0.89-0.98 | <0.001 |  | 0.78 | 0.45-0.91 | <0.001 |
| pLASRcd, s^-1^ | 0.86 | 0.66-0.95 | <0.001 |  | 0.85 | 0.61-0.94 | <0.001 |
| pLASRct, s^-1^ | 0.90 | 0.74-0.96 | <0.001 |  | 0.91 | 0.78-0.97 | <0.001 |
| RASr, % | 0.89 | 0.71-0.96 | <0.001 |  | 0.80 | 0.51-0.92 | <0.001 |
| RAScd, % | 0.83 | 0.58-0.93 | <0.001 |  | 0.81 | 0.51-0.92 | <0.001 |
| RASct, % | 0.97 | 0.92-0.99 | <0.001 |  | 0.84 | 0.59-0.94 | <0.001 |
| pRASRr, s^-1^ | 0.91 | 0.77-0.97 | <0.001 |  | 0.83 | 0.56-0.93 | <0.001 |
| pRASRcd, s^-1^ | 0.95 | 0.88-0.98 | <0.001 |  | 0.77 | 0.42-0.91 | 0.001 |
| pRASRct, s^-1^ | 0.88 | 0.70-0.95 | <0.001 |  | 0.85 | 0.62-0.94 | <0.001 |

Abbreviations as in Supplementary Table 1.
